# Exosomal microRNAs: Pleiotropic Impacts on Breast Cancer Metastasis and Their Clinical Perspectives

**DOI:** 10.3390/biology10040307

**Published:** 2021-04-07

**Authors:** Li-Bo Tang, Shu-Xin Ma, Zhuo-Hui Chen, Qi-Yuan Huang, Long-Yuan Wu, Yi Wang, Rui-Chen Zhao, Li-Xia Xiong

**Affiliations:** 1 Department of Pathophysiology, Basic Medical College, Nanchang University, Nanchang 330006, China; 6300817522@email.ncu.edu.cn (L.-B.T.); 6301616056@email.ncu.edu.cn (Q.-Y.H.); 6300817039@email.ncu.edu.cn (L.-Y.W.); 401442719031@email.ncu.edu.cn (Y.W.); jp6300517180@qmul.ac.uk (R.-C.Z.); 2 Second Clinical Medical College, Nanchang University, Nanchang 330006, China; 6300817314@email.ncu.edu.cn; 3 Queen Mary School, Jiangxi Medical College, Nanchang University, Nanchang 330006, China; 6300517146@email.ncu.edu.cn; 4 First Clinical Medical College, Nanchang University, Nanchang 330006, China; 5 Jiangxi Province Key Laboratory of Tumor Pathogenesis and Molecular Pathology, Nanchang 330006, China

**Keywords:** exosomal microRNA, breast cancer, metastasis, cancer stem cell, angiogenesis, chemoresistance, prognosis

## Abstract

**Simple Summary:**

This review has comprehensively summarized the most recent studies in the last few years about exosomal microRNAs on metastasis of breast cancer (BC), systematically outlined and elucidated the pleiotropic roles that exosomal microRNAs take, and discussed the specific underlying mechanisms related. Besides, we also clearly demonstrate the clinical implications of exosomal microRNAs in various aspects, including early-stage discovery of BC, systematic and targeted therapy, and the selection of anti-cancer chemo-agents. This review clarifies the scope and extent of current studies about the relationship between exosomal microRNAs and metastasis of BC, laying a solid foundation for further laboratory studies. What’s more, it links the theoretical researches and clinical features about exosomal microRNAs on metastasis of BC together, providing novel clues for BC diagnosis, chemotherapeutics and drug efficacy evaluation in clinical, which indicates the great potential of exosomal microRNAs in clinical significance.

**Abstract:**

As a major threat factor for female health, breast cancer (BC) has garnered a lot of attention for its malignancy and diverse molecules participating in its carcinogenesis process. Among these complex carcinogenesis processes, cell proliferation, epithelial-to-mesenchymal transition (EMT), mesenchymal-to-epithelial transition (MET), and angiogenesis are the major causes for the occurrence of metastasis and chemoresistance which account for cancer malignancy. MicroRNAs packaged and secreted in exosomes are termed “exosomal microRNAs (miRNAs)”. Nowadays, more researches have uncovered the roles of exosomal miRNAs played in BC metastasis. In this review, we recapitulated the dual actions of exosomal miRNAs exerted in the aggressiveness of BC by influencing migration, invasion, and distant metastasis. Next, we presented how exosomal miRNAs modify angiogenesis and stemness maintenance. Clinically, several exosomal miRNAs can govern the transformation between drug sensitivity and chemoresistance. Since the balance of the number and type of exosomal miRNAs is disturbed in pathological conditions, they are able to serve as instructive biomarkers for BC diagnosis and prognosis. More efforts are needed to connect the theoretical studies and clinical traits together. This review provides an outline of the pleiotropic impacts of exosomal miRNAs on BC metastasis and their clinical implications, paving the way for future personalized drugs.

## 1. Introduction

Breast cancer (BC) is one of the most common diseases among women in contemporary society and shows a high mortality rate. In 2018, there are about 2.1 million new cases of BC in the world, accounting for nearly a quarter of female cancer cases. BC is the most commonly diagnosed cancer in the vast majority (83%) of countries, and it is also being the main cause of cancer death in more than 100 countries [1]. As a critical program in BC carcinogenesis, metastasis is attributed to the majority of deaths in people suffering from BC. Metastasis is a complicated and multi-step process, which involves epigenetic and genetic mutations, patterns of dissemination and disaggregation, chaotic and irregular angiogenesis, invasion-metastasis cascades, the epithelia-mesenchymal transition (EMT), and stemness maintenance of cancer stem cells (CSCs). Any treatment targeting molecules in these pathological processes can affect the progress of metastasis [2]. 

Currently, metastatic disease is regarded to be incurable due to specific characteristics which define it. Some novel traits derived from the metastatic lesions make it more malignant than the original one. This discordance in tumor features of metastatic lesions becomes a great obstacle to the multiple clinical treatments. Metastatic tumor cells generally acquire high genetic instability. Extensive dis-regulation in the genomics repair and transmission processes drives to the high level of genetic mutations. Monogenetic and polygenetic clones are the two major ways in the formation of tumor subpopulations. During the process of cloning, different subpopulations may acquire segregated anatomical distributions and inherit diverse genetic advantages. This molecular discordance can explain the heterogeneity in the biological behavior of metastatic groups. In clinical, the changes in the expression profiles of metastasis groups hide the validated targets for some specialized therapy. And the newly-activated signaling pathways in subpopulations also make the existing medicine less effective. Besides, it is thought that metastases partly evolve from the dissemination of cancerous cells with stemness. While stemness-like properties can impart chemotherapy resistance to cells. What is more, there is another malignant tumor subpopulation, called dormant tumor cells. These cells can stay in a latent state, escape from the initial treatments and awake later. The heterogeneity of metastatic cells. Though some clinical therapies have successfully managed the primary BC lesions, no effective strategies are capable to control relapses and cope with BC metastases [3].

Based on molecular features, BC can be divided into 4 groups, luminal A, luminal B, human epidermal growth factor receptor 2 (HER2), and basal type. Well-established classification contributes to a more accurate diagnosis and the improvement of therapy selection. For patients with luminal A or B type BC, they will bring about a better prognosis after receiving endocrine-based therapy or standard schedule chemotherapy [4]. About HER2-positive patients, anti-HER2 immunotherapy such as targeted antibodies (e.g., trastuzumab/Herceptin) can exert rapidly and sustainedly tumor-inhibitory effects [5]. The clinical features of triple-negative breast cancer (TNBC) appear no expression of estrogen receptors (ER), HER2, and progesterone receptors (PR) on the surface of cancerous cells. The absence of characteristic biomarkers makes endocrine-related therapy not applicable for TNBC therapy. As a novel clinical paradigm for systemic therapy, targeted immunotherapy combined with chemotherapy still shows limited efficacy and deleterious effects on healthy organs in the vicinity. Nanotechnology, an emerging tool, works to artificially design some specific nanoparticles with ideal structures, functions, and properties to satisfy the clinical application. Several products from nanotechnology, such as conjugated dendrimers and modified micelles, possess great prospects in copying with TNBC and can be considered as a new direction to make a breakthrough in BC treatment [6].

**Figure 1:**
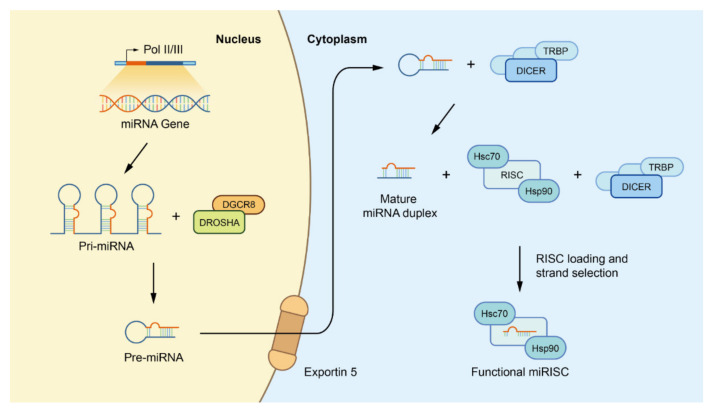
Canonical biogenesis of miRNAs. Firstly, miRNA genes are transcribed as primary precursors (pri-miRNA) by the action of RNA polymerase and further processed by the Drosha complex, which consists of one ribonuclease called Drosha and two molecules of the RNA-binding protein DGCR8, to form pre-miRNAs. Subsequently, pre miRNAs are exported to the cytoplasm by exportin-5 at the nuclear envelope before being cleaved by the Dicer/TRBP complex to form mature miRNA duplexes. Finally, with the help of the Dicer/TRBP complex and HSC70/HSP90 chaperone proteins, one strand of the miRNA duplex is placed into the RISC to form a functional miRISC for subsequent regulation. Abbreviations: miRNAs: microRNAs; pri-miRNA: primary miRNA; DGCR8: DiGeorge syndrome critical region 8; pre-miRNAs: precursor miRNAs; TRBP: transactivation response RNA binding protein; HSC70: heat shock cognate protein 70; HSP90: heat shock cognate protein 90; RISC: miRNA induced silencing complex.

Exosomes, with a featured ‘saucer-like’ morphology, are membrane-enclosed vesicles originally made inside the intracellular multivesicular endosomes and multivesicular bodies (MVBs) by invagination and budding [7]. Exosome vesicles contain cytosol and coat the extracellular domain of transferrin receptors at their surface. There are many highly-conserved proteins and lipids in rafts in exosomes with various functions, such as antigen presentation, integrins, and immunoglobulin-family members [8]. Besides, the lumen of exosome has uncovered different nucleic acids, including messenger RNAs (mRNAs), microRNAs (miRNAs), and other non-coding RNAs (ncRNAs). MiRNAs are endogenously small noncoding RNA gene products that commonly regulate mRNA expression post-transcriptionally by repressing translation and/or transcript decay [9]. The biological synthesis of exosomal miRNAs in cells is pictured below in Figure 1. MiRNAs carried in exosomes are not randomly assigned [10]. In addition, their expression levels are not constant in different conditions [11]. More researches have garnered attention for the roles of miRNAs in exosomes.

Commonly, exosomes can be excreted from various cell types, such as smooth muscle cells, endothelial cells, and immunocytes [12,13]. When exosomes circulate in the body fluids, they can be picked up by neighboring cells or distant cells, and subsequently, regulate their biological functions [14]. Several studies have reported that miRNA can take an important part in cell function modification, immunity regulation, and tumorigenesis. For example, miRNAs, as paracrine mediators, are in a relatively high abundance of cardiac fibroblast-derived exosomes, stimulating cardiac remodeling, and inducing cardiomyocyte hypertrophy [15]. Migration and angiogenesis in the human dermal microvascular endothelial cells-1 (HMEC-1) line can be boosted by exosomal miR-214 from HMEC-1 cells nearby [16]. Exosomal miR-21 and miR-29a are able to act as ligands for toll-like receptors (TLRs) and promote the activation of immunocytes [17]. MiR-92 and miR-25-3p contained in plasma exosomes and total blood exosomes of breast cancer patients (BCPs) can induce angiogenesis and epithelial-mesenchymal transition (EMT), and increase the total migration path length of BC cells. 

Since the quantity and ingredients of exosomal miRNAs are different between physiological and pathological conditions, its clinical application is also a field worth exploring. Exosomal miRNAs can be used as a marker for disease diagnoses [18], such as glioblastoma [19], chronic obstructive pulmonary disease [20], colorectal cancer [21], prostate cancer [22], and BC [23]. Emerging evidence suggests that tumor-derived (TD) exosomes provide a soil conducive to tumor growth by mediating communication within the tumor microenvironment, i.e., exosomal miR-105 released from the BC cell lines MCF-10A and MDA-MB-231 dissolve endothelial cells identity by weakening Zonula occludens 1 (ZO-1) gene expression which promotes metastases of cancer daughter cells to the lung and brain [24]. Thus, TD-exosomes can be considered as targeted therapies. Specificity, accessibility from the extracellular domain, and stability make exosomes effective markers for tumor diagnosis, treatment, and prognosis [25]. 

**Figure 2:**
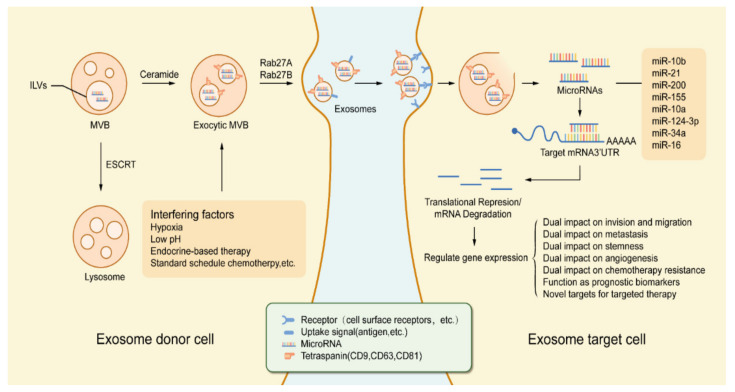
Tumor regulation process based on exosomal miRNA in the tumor microenvironment. Exosomes are derived from MVB which germinate inward in the form of ILVs. The formation of exosomes as well as the sorting of cargo into lysosomes involves the ESCRT. In addition, exosome production and secretion are also completed by a non-ESCRT dependent process that involves the sphingolipid ceramide and the enzyme neutral sphingomyelinase. Stimulation by various types of chemical, physical factors can induce the secretion of exosomes, such as hypoxia, low pH, endocrine therapy, etc. After the fusion of MVBs with the cell membrane, exosomes are secreted into the extracellular space under the action of Rab GTPases (Rab27A, Rab27B). Exosomes released from exosome donor cells (tumor cells, etc.) will exhibit similar receptor ligands or antigens as their cells of origin. The endocytic process of exosomes occurs with the activation of receptors or bioactive lipid ligands at the cell surface. After exosomes are endocytosed by exosome target cells (endothelial cells, etc.), miRNAs in exosomes are released. Transferred miRNAs are bioactive and can regulate gene expression in exosomal target cells through post-translational regulation of mRNA expression, which in turn leads to mRNA instability or degradation. miRNA-dependent gene regulation can affect various types of processes involved in tumorigenesis and development, and changes in gene expression levels can also be used as a marker for judging tumorigenesis and progression. Abbreviations: MVB: multivesicular body; ILVs: intraluminal vesicles; ESCRT: endosomal sorting complex required for transport; Rab27A: Ras-associated binding 27A; Rab27B: Ras-associated binding 27B.

In this study, as shown in Figure 2, we reviewed the latest research progress in this field. We uncovered that exosomal miRNAs not only take part in enhancing cell growth and variance of EMT or MET but also influence the local invasiveness and metastasis of BC cells. Besides, exosomal miRNA can modify tumor immunogenicity, active angiogenic switch, escape programmed apoptosis as well as deliver drug resistance. By consulting numerous kinds of literature, here we discussed the underlying molecular mechanisms involved in these pathological processes, which contribute to the prevention, diagnosis, treatment, and prognosis of BC. How the signaling pathways involved by exosomal miRNAs regulate metastasis of BC cells is the highlight of this review.

## 2. Exosomal microRNAs That Enhance Aggressiveness of BC Cells

### 2.1. Exosomal microRNAs That Promote Invasion and Migration of BC Cells

#### 2.1.1. miR-21

Early studies have shown that exosomal miR-21 is overexpressed in different types of tumor tissues, including BC [26,27]. It is involved in almost all stages of BC development [28].

The knockdown of exosomal miR-21 can inhibit the invasion and migration of BC cells. On the contrary, the up-regulation of exosomal miR-21 inhibits the expression of the gene of phosphate and tension homology deleted on chromosome ten (PTEN), which suppresses the constitutive activation of downstream components of the phosphatidylinositol 3-kinase (PI3K) pathway, thereby suppressing the expression of the tumor suppressor gene Drg-1 (differentiation-related gene 1) [29].

Besides, several regulatory mechanisms of exosomal miR-21 in BC cells have been proposed by recent studies. They suggested that miR-21 can directly inhibit the expression of the corresponding tumor suppressor by directly targeting the 3’UTR of mammary serine protease inhibitor (maspin), programmed cell death protein 4 (PDCD4), or tumor suppressor gene tropomyosin 1 (TPM1) [30]. Yin et al. found that maspin interacts with the uPA associated with the cell surface and stimulates the internalization of the uPA/uPAR complex [31]. On the other hand, PDCD4 can down-regulate urokinase-type plasminogen activator receptor (uPAR) through its promoter region [32]. uPAR is a promoter of cell invasion and metastasis [33]. The reduction of uPAR level caused by maspin or PDCD4 can significantly inhibit the invasion and migration ability of BC cells. Interestingly, PDCD4 can also inhibit the proliferation and invasion of BC cells by interacting with eukaryotic initiation factor 4A (EIF4A), participating in the down-regulation of mitogen-activated protein kinase kinase kinase kinase 1 (MAP4K1) transcription, and up-regulating p21 to inhibit the transcription of cyclin-dependent kinase 1 (CDK1)/cdc2 [34,35]. For TPM1, Zhu et al. discovered that overexpression of TPM1 inhibits the invasion of BC cells, but the specific mechanism remains to be investigated [30].

#### 2.1.2. miR-10b

Exosomal miR-10b is closely linked to BC size, progression, and degree of invasion [36]. Previous research on BC cells found that exosomal miR-10b can directly inhibit the translation of Homeobox D10 (HOXD10) for promoting the migration of BC [37]. HODX10 is an mRNA encoding a transcriptional repressor, which reduces the expression of some genes involved in extracellular matrix (ECM) remodeling and cell migration, such as rat sarcoma (Ras) homolog gene family member C (RHoC), Membrane-type 1 matrix metalloproteinase (MT1-MMP), a3-integrin, and urokinase plasminogen activator receptor [38]. In addition, studies have confirmed that after inhibiting the expression of TBX5, the up-regulation of exosomal miR-10b levels simultaneously causes the down-regulation of PTEN and another tumor suppressor, Dual-specificity tyrosine phosphorylation-regulated kinase 1A (DYRK1A), which ultimately affects BC invasion and migration [39,40]. Currently, based on the regulatory relationship between sphingolipid ceramide and exosomes, the researchers found that the secretion of exosomal miR-10b is also ceramide-dependent regulation [41]. It is worth noting that exosomal miR-10b can act as a sponge for syndecan-1, and significantly affect the migration and invasion of BC through the miR-10b/syndecan-1 axis. In terms of mechanism, it may be achieved by the activation of miR-10b/syndecan-1 axis that leads to ras homologous oncogenes guanosine triphosphatases (Rho-GTPase)-dependent regulation of cytoskeletal function and down-regulation of E-cadherin expression [42].

#### 2.1.3. miR-1246

MiR-1246, a 25-nt miRNA that is situated on chromosome 2q31.1, originates from U2 small nuclear RNA (RNU2-1) transcript degradation [43]. Studies have shown that exosomal miR-1246 is significantly upregulated in BC [44]. Interestingly, Li et al. found that exosomal miR-1246 is over-expressed in metastatic BC MDA-MB-231 cells, and can also inhibit the expression level of its target gene Cyclin-G2 (CCNG2) to promote BC cell invasion [45]. These findings provide a basis for targeting exosomal miR-1246 to interfere with BC.

#### 2.1.4. miR-373

Exosomal miR-373 has been determined to be functionally related to many biological processes, such as cell proliferation, migration, invasion, and apoptosis of tumors, including BC. Previous studies have found that exosomal miR-373 is highly correlated with BC invasion and migration, especially high-invasive BC [46]. By establishing the MCF-7 cell line, Huang et al. confirmed that exosomal miR-373 promotes cell migration and invasion, at least in part, by limiting CD44 expression directly [47]. In addition, it has recently been reported that exosomal miR-373 may be a plasma-based biomarker that can be used to indicate the lymph node status of BC [48]. These findings provide new ideas for further research on the relationship between exosomal miR-373 and BC aggressiveness.

#### 2.1.5. miR-17-5p

Exosomal miR-17-5p specifically targets HMG-box protein 1 (HBP1)/Wnt/β-catenin signaling cascade to enhance the migratory and invasive ability of BC cell lines. HBP1 serves as a cell-cycle suppressor and oncogene transcriptional suppressor. By controlling the transcriptional actives governed by T-cell factor/lymphoid enhancer-binding factor (TCF/LEF), HBP1 carefully monitors the Wnt/β-catenin signaling network. HBP1-mediated inhibition of exosomal miR-15-5p facilitates the acquisition of BC cells to more malignant phenotypes, as well as invasion and migratory ability [49]. It is worth noting that Lv et al. recently compared the levels of exosomes miR-17-5p in the serum of 83 BC patients and 34 healthy women and found an obvious decrease in the serum of BC patients decreased [50]. These results are consistent with previous studies and strongly suggest that exosomal miR-17-5p can be used as a new diagnostic biomarker for BC.

#### 2.1.6. miR-96

Protein tyrosine phosphatase non-receptor type 9 (PTPN9), an intracellular phosphatase, governs the pro-migratory and pro-invasive functions of several signal molecules, such as epidermal growth factor receptor (EGFR), signal transducer and activator of transcription 3 (STAT3), and human epidermal growth factor receptor-2 (ErbB2). Exosomal miR-96 can specifically recognize and binding to PTPN9 mRNA and cutting off subsequent expression activities, which exerts a promotive effect on non-transform cells for the acquisition of increased motion ability [51]. 

#### 2.1.7. miR-106b

The content of exosomal miR-106b is particularly abundant in cancerous cells and the plasma of BC patients. BC cells exposed by exosomal miR-106b obtain the increased motion ability. Involved in numerous cellular metabolic activities, restoration of the expression of fucosyltransferase 6 (FUT6) can counteract the oncogenic effects exerted by exosomal miR-106b. Moreover, re-expression of exosomal miR-106b apparently down-regulates the content of FUT6, suggesting that exosomal miR-106b plays the pro-migrative and pro-invasive roles by diminishing the expression of FUT6 [52].

### 2.2. Exosomal microRNAs That Promote Distant Metastasis of BC Cells

#### 2.2.1. miR-10b

In recent years, exosomal miR-10b has been one of the most thoroughly studied miRNAs. Alongside invasion and migration of BC cells, exosomal miR-10b participates in metastasis of BC as well [39]. Mounting evidence has shown a highly positive correlation between exosomal miR-10b and distant metastasis, as well as lymph node metastasis of BC [53].

Epigenetic signals from the tumor microenvironment regulate the transition of BC cells from dormancy to metastatic growth [54,55]. In addition to exploring crosstalk studies on BC cells themselves, the latest research has investigated the role of exosomal miR-10b in the tumor microenvironment. By establishing an exosome-free culture model of metastatic MDA-MB-231 cells, Ma et al. found that overexpression of miR-10b in MDA-MB-231 cells would lead to an increase in the extracellular level of miR-10b (i.e., exosomal). Exosomal miR-10b can be effectively absorbed by a variety of receptor cells, such as MCF-10A, HEK-239T, and MCF-7 cells [41,56]. Additionally, in the non-metastatic human BC cell line SUM159, the absorption of exosomal miR-10b leads to more pronounced lung metastases and macroscopic peritoneal metastases [56].

Interestingly, after the first identification of the Twist/miR-10b/HOXD10/RHoC signaling pathway, it is demonstrated as a participant when exosomal miR-10b regulates BC metastasis [57,58,59]. Notably, another study found that loss of E-cadherin during BC progression leads to increased expression of exosomal miR-10b. At this point, exosomal miR-10b binds to neurofibromatosis type 1 (NF1) and HODX10 to activate the Rho-associated kinase (ROCK)/c-Jun signaling pathway, thereby promoting the invasion and metastasis of BC cells [60].

#### 2.2.2. miR-503

Xing et al. found that miR-503 is one of the miRNAs with the highest expression in the exosomes of MCF-7 cells with X-inactive specific transcript (XIST) knockdown. They observed that serum levels of exosomal miR-503 in patients with brain metastases from BC are significantly increased. Mechanistically, exosomal tumor cells-derived miR-503 promotes the transformation of microglia from M1 to M2 phenotypes by manipulating STAT3 and NF-κB pathways. Subsequently, activation of these signaling pathways enhanced programmed cell death ligand 1 (PD-L1) expression to suppress local immunity, thus promoting brain metastasis of BC cells [61]. Based on the results above, we suggest exosomal miR-503 is a tumor-promoting exosomal miRNA that endows potential brain metastasis ability to BC cells.

#### 2.2.3. miR-122

Abnormally activated anaerobic glycolysis to reprogram energy metabolism, called the “Warburg effect”, is considered a hallmark of cancer [62]. Quite abundant in all BC cell lines, exosomal miR-122 can be a candidate molecule to reflecting the metastatic phase in the early stage of BC progression. Overexpression of exosomal miR-122 speeds the spontaneous transformation from in situ ductal carcinoma lesions to malignant tumors. Restraining glucose metabolism, exosomal miR-122 inhibits primary lesion growth while functions as a signaling molecule to modify the pre-metastatic microenvironment, and making adequate preparation for further metastatic colonization. After receiving exosomal miR-122, the pyruvate kinase (PKM) involves in glycolysis and glucose transporter 1 (GLUT1), a transporter for glucose uptake is downregulated in niche cells, altering the metabolic rate and energy metabolism. The sufficient energy supply is hijacked by BC cells, building a pro-growth microstructure, and facilitating more metastases occurrence [63].

#### 2.2.4. miR-200

It is proposed that Sec23a facilitates the release of metastasis-suppressive substrates, such as Tinagl1 and Igfbp4. Exosomal miR-200 can directly inhibit the functions of Sec23a to fuel the formation of metastatic subpopulations [64]. Besides, miR-200 is able to downregulate Yes-associated protein 1 (YAP1), which subsequently motivates metastasis efficiently [65].

#### 2.2.5. miR-105

Exosomal miR-105 is specifically enriched in metastatic BC cells, reflecting the progressing of metastasis in early-stage BC. Zonula occludens 1 (ZO-1), a tight junction protein, is the major fundamental molecule composing the intercellular adhesion molecules of epithelial and endothelial cells. Expression of exosomal miR-105 can downregulate the content of ZO-1 in both mRNA and protein levels. Transfected with exosomal miR-105, BC cells show a significant deficiency in ZO-1 and recycle occludin, another functional factor from cell junction. Released from metastatic cells, exosomal miR-105 alters vessel integrity, augments vessel permeability as well as promotes neovessel sprouts, facilitating cancerous cells to seed in anywhere good for further oncogenesis [24].

#### 2.2.6. miR-21

Exosomal miR-21 promotes oncogenic progress in numerous cancers, also motivating metastasis of BC cells. Downregulation of exosomal miR-21 attenuates cell motion and metastatic colonization [66]. As a tumor-suppressor gene, leucine zipper transcription factor-like 1 (LZTFL1) regulates metastasis by blocking β-catenin translocation to the nucleus and activating pro-metastatic gene expressions, such as slug and snail. Targeting LZTFL1, exosomal miR-21 removes the inhibition of LZTFL1 to β-catenin, upregulating EMT marker, and metastatic-dependent functional proteins. MiR-21/LZTFL1/β-catenin/EMT axis effectively fuels metastasis of BC cells [67].

## 3. Exosomal microRNAs That Attenuate Aggressiveness of BC Cells

### 3.1. Exosomal microRNAs That Inhibit Invasion and Migration of BC Cells

#### 3.1.1. miR-564

The mitogen-activated protein kinase (MAPK) and PI3K signaling pathways are always hijacked in BC, associated with independent cell growth, EMT, and migration [68]. Exosomal miR-564 impairs cell proliferation, viability, and motility by directly modifying the expression of a cohort of related genes, Akt2, guanine nucleotide-binding protein alpha-12 (GNA12), glycogen synthase 1 (GYS1), and SRF, thereby dually inhibiting MAPK and PI3K pathway and causing irreversible cell-cycle arrest. Acting as an oncogenic suppressor, the expression of exosomal miR-564 effectively blocks the migration and invasion and related pathological process of BC cells [69].

#### 3.1.2. miR-10a

Aberrantly activated PI3K/Akt/mTOR pathway is commonly found in various cancers. BC is no exception. Exosomal miR-10a reduces the expression of the mechanistic target of rapamycin (mTOR), PI3K, and Akt by specifically targeting a key molecule PI3K associated with this pathway. By interfering with the function of this pathway, exosomal miR-10a weakens the migration and motility of BC cells [70].

#### 3.1.3. miR-34c

Metastatic cells selectively downregulate the expression of exosomal miR-34c. After the transfection of exosomal miR-34c, the migration and invasion of BC cell lines are inhibited G-protein-coupled receptor kinase interacting protein-1 (GIT1) exerts a regulatory role in intercellular adhesion and migration. In addition, it also facilitates the stabilization of Paxillin, a fundamental molecule in adhesion. Tao et al. demonstrated that overexpression of exosomal miR-34c can significantly inhibit the invasion and migration [71].

#### 3.1.4. miR-217

As mentioned earlier, miR-217 is considered as a tumor promoter, while some found exosomal miR-217 can inhibit the proliferation and invasion of a variety of cancers, such as pancreatic ductal adenocarcinoma [72], liver cancer [73], and esophageal squamous cell carcinoma [74]. BC is no exception. Interestingly, Zhou et al. discovered that exosomal miR-217 targets krüppel-like factor 5 (KLF5), a powerful carcinogen in both HCC1937 and HCC1806 cell lines, to inhibit the development of TNBC [75]. This supplies the feasibility for the treatment of TNBC through the miR-217/KLF5 axis.

Coincidentally, KLF5 is only highly expressed in HCC1937 and HCC1806 but is low in MCF-7, MDA-MB-231, and SKBR3. Additionally, exosomal miR-217 does not inhibit the expression of DACH1 and PTEN in HCC1937 and HCC1806 cell lines as it did in other cells such as MCF-7 [75]. Therefore, we suggest that the specific role of exosomal miR-217 in BC may be related to cell line specificity.

#### 3.1.5. miR-100

Exosomal miR-100 is a pleiotropic molecule. Wnt/β-catenin signaling pathway is a widely-studied pathway. It takes part in numerous biological processes, including cell proliferation and cell motility, also contributing to tumorigenesis, such as invasion and metastasis. Exosomal miR-100 can repress the expression of pro-invasive and oncogenic factors such as matrix metalloproteinase-7 (MMP-7), cellular-myelocytomatosis viral oncogene (C-myc), and FZD-8. Moreover, it can also upregulate inhibitory molecules such as glycogen synthase kinase 3 (GSK3) belonging to the Wnt/β-catenin signaling pathway to imparting the motion tendency of MDA-MB-231 BC cells [76,77]. Homeobox A1 (HOXA1), an oncogene motivating tumor growth and tumor motility by regulating the expression of downstream pro-migration and pro-invasion genes, cyclin D1 (CCND1), MET, smoothened (SMO), and semaphorin 3C (SEMA3C). Jiang et al. revealed in their research that HOXA1 is blocked by exosomal miR-100 to impair the aggressive phenotypes of BC cell lines [77].

#### 3.1.6. miR-19b-3p, miR-19a-3p, and miR-1226-3p

Aquaporins (AQPs) selectively facilitate the transport of water between different body compartments to maintain homeostasis. AQP3 and AQP5 are quite abundant in triple-negative BC patients. Overexpressed AQP5 in epithelial cells prompts cell detachment and migration, which contributes to invasiveness and metastasis of tumors. AQP5 also regulates the migration of BC. Experiments showed that exosomal miR-19a-3p, miR-19b-3p, and miR-1226-3p are metastatic regulators that exert their roles by regulation of target gene expression, including AQP5 [78]. 

Exosomal miR-19b-3p blocks the process of transcription by directly binding to AQP5 mRNA. Exosomal miR-19a-3p may bind to AQP5 mRNA at a site different from exosomal miR-19b-3p to inhibit AQP5 expression. Alternatively, it indirectly regulates AQP5 expression by modulating genes that can influence AQP5 expression. For exosomal miR-1226-3p, it decreases the cellular content of AQP5 by motivating the degradation of AQP5 mRNA. So an obvious reduction in migration occurs in MDA-MB-231 cells transfected with these three miRNAs respectively due to the diminished expression of AQP5 [78]. 

Besides, exosomal miR-19a-3p diminishes the content of Fos-related antigen 1 (FRA-1) which prompts the invasiveness and tumor malignancy of BC cells, by weakening the expression level of the FOS like 1 (FOSL1) gene coding for it. Notably, exosomal miR-1226-3p can reduce mucin 1 expression in BC cells by modifying mucin 1 mRNAs [79]. These exosomal miRNAs provide new ideas for controlling the invasion and metastasis of BC in various ways in the future.

#### 3.1.7. miR-148a and miR-148b-3p

Decreased expression of exosomal miR-148b-3p occurs in various kinds of BC cell lines, which are largely associated with poor prognoses in BC [80]. Tripartite motif-containing 59 (TRIM 59), a major target gene of miR-148b-3p that is highly expressed in BC. Western blot analysis demonstrated that the up-regulation of TRIM 59 expression increased the abundance of EMT-related proteins in MDA-MB-231 cells while inhibiting the levels of apoptosis-related proteins. This reveals the role of TRIM 59 in promoting tumor growth, improving tumor migration and invasion, and playing an anti-apoptotic effect. MiR-148b-3p carried by exosomes derived from human umbilical cord mesenchymal stem cells (HUCMSCs) reduced viability of BC, restrains tumor progression, and weakens tumor malignancy by down-regulating the expression level of TRIM 59 [81]. Therefore, the subsequent oncogenic pathways and the effects in tumorigenesis are almost blocked by exosomal miR-148b-3p [81]. Moreover, previous studies showed that overexpressed exosomal miR-148a takes part in inhibiting tumor motility, including invasion and migration of MCF-7 and MDA-MB-231 BC cells [82]. Interestingly, the latest research also proved that the exosomal miR-148a in BC patients is significantly reduced, along with the adverse outcome of BC [83].

#### 3.1.8. miR-503

Ander varying circumstances, as a pleiotropically-functional factor carried by exosomes from cancerous tissues and endothelial cells, miR-503 may exert opposite effects on the invasiveness of BC cells. As mentioned before, exosomal miR-503 derived from BC cell lines reinforces migration. However, a few studies surprisingly provide new insight into exosomal miR-503 as a metastasis-suppressing exosomal miRNA in BC. Several studies have found exosomal miR-503 from endothelial cells impairs the invasive capacity [61,84]. As a powerful effector, exosomal miR-503 negatively regulates the expression of cyclin D2 (CCND2) and cyclin D3 (CCND3) in both RNA and protein levels. The subsequent impact on BC cells of this interaction is to apparently reduce proliferation rate and diminish invasive capacity [84].

In summary, we cannot conclude whether exosomal miR-503 is a tumor-promoting or suppressing exosomal miRNA; numerous unknown events remain to be discovered to explore the possible explanation for the dual effects of exosomal miR-503 in BC.

#### 3.1.9. miR-17/20

MicroRNA17/20 abundance is reduced in highly invasive BC cell lines and node-positive BC specimens. Serving as tumor-suppressive miRNA, exosomal miR-17/20 blocks irregular cell-cycle by silencing E2F transcription factor 1 (E2F1) expression, as well as causes alteration in the secretion of a series of pro-metastatic cytokines, such as C-X-C motif chemokine ligand 1 (CXCL1), cytokeratin 8 (CK8), and Interleukin-8 (IL-8). By directly binding to IL-8 mRNA, exosomal miR-17/20 impairs the transcription activities of the IL-8 gene. Plasmin, derived from plasminogen, is crucial in the reinforcement of cell motility and metastasis for its role in degrading ECM and is activated by alpha-enolase (alpha-ENO) and CK8 bounce. Exosomal miR-17/20 represses the function of CCND1 to block the activity of CK8 and indirectly reduces alpha-ENO expression. By modifying pro-malignant cytokines excretion and inhibiting plasmin-activator, exosomal miR-17/20 governs BC pre-metastatic behavior [85].

### 3.2. Exosomal microRNAs That Inhibit Distant Metastasis of BC Cells

#### 3.2.1. miR-429

Exosomal miR-429 is a migration suppressor, belonging to the miR-200 family. As two candidate molecules of exosomal miR-429, zinc finger E-box binding homeobox 1 (ZEB1) and V-crk sarcoma virus CT10 oncogene homolog-like (CRKL) cooperate with other fundamental molecules and govern a series of signal cascades to enhance migratory capacity. Mechanistically, exosomal miR-429 reduces the content of ZEB1 and CRKL at the protein level to interrupt the subsequent signal networks [86]. Another in vitro experimental study uncovered the inhibitory roles of exosomal miR-429 in bone metastasis. Cancerous cells transfected with miR-429 show the decreased expression level of CrkL and matrix metalloproteinases-9 (MMP-9), as well as reduced penetration in the local bone microenvironment and fewer metastatic lesions [87]. These indicate the miR-429/CrkL/MMP-9 axis is capable to alter the tendency of cancerous cells to seed in the bone microenvironment.

#### 3.2.2. miR-124-3p

Compared with adjacent tumor-free tissues, the content of exosomal miR-124-3p is remarkably reduced in former BC tissues [88]. Exosomal miR-124-3p suppresses the EMT processes by upregulating the protein level of E-cadherin and downregulating that of N-cadherin and Vimentin. By directly targeting programmed cell death protein 6 (PDCD6), exosomal miR-124-3p impairs the migratory and motility capacity of BC cells, reducing metastasis [89]. The decline of exosomal miR-124-3p is largely associated with lymph node metastasis and a poorer survival rate [88].

#### 3.2.3. miR-31

Exosomal miR-31 is a pleiotropic molecule taking part in several steps of metastasis and is always specifically diminished or deleted in malignant BC cells [90]. The maintenance of exosomal miR-31 functions facilitates the prevention of normal epithelial cells and tumor cells from obtaining a more aggressive phenotype, such as chemoresistance. By attenuating the expression of a series of pro-metastatic genes, such as frizzled class receptor 3 (Fzd3), c-matrix metalloproteinase-16 (cMMP16), myosin Phosphatase-Rho interacting protein (M-RIP), integrin alpha 5 (ITGA5), hexahydro-1,3,5-trinitro-1,3,5-triazine (RDX), and Ras homolog gene family member A (RhoA), exosomal miR-31 weakens metastatic and metastatic-dependent traits. Gain- and loss-of-function assays dictate that exosomal miR-31 reduces the number of metastatic colonization and restrains the size of the lesion, as well as blocks the early stage of the post-intravasation cascade, including intravasation, intra-capillary viability, and penetration, creating an unfavorable environment for cancer development [91]. The discovery of exosomal miR-31 provides new insights for the treatment and prevention of BC.

#### 3.2.4. miR-124

A number of studies have uncovered the roles played by exosomal miR-124 in the oncogenic process of various malignant cancers, such as cervical cancer, prostate cancer, and BC, demonstrating its tumor-suppressive function [92]. Impairing exosomal miR-124 imparts a more pathological feature of BC cells. Clinically, the low level of exosomal miR-124 contributes to the increased lesion seeding in the bone, rapid appearance of metastasis in bone, and poorer survival free from bone-metastasis [93]. Furthermore, exosomal miR-124 modifies the tumor-microenvironment to weaken the communication between cancerous cells and the bone microenvironment. Interleukin-11 (IL-11), a tumor-motivator, augments the activities of the GP130-Janus kinase network to fuel the tumorigenesis of epithelial tumors, including BC. Excreted by BC cells, IL-11 enhances osteoclastogenesis by facilitating the supplement of osteoclast progenitor cells. Cai et al. have found that exosomal miR-124 exerts its metastasis-inhibitory influence on BC cells by inhibiting IL-11 expression, which significantly suppresses the proliferation and survival of osteoclast progenitor cells [93].

#### 3.2.5. miR-1

Exosomal miR-1 is often found downregulated in BC and associated with worse overall survival. Restoration of the function of BC contributes to the blockage of cell growth, motion, and metastasis. Mechanically, exosomal miR-1 targets Frizzled 7 and TNKS2 to disturb the Wnt/β-catenin signaling cascades and activates Bcl-2, which impairs metastasis and subsequent tumorigenesis [94,95].

#### 3.2.6. miR-193a

Deficiency in exosomal miR-193a activities is common in several cancers [96]. Exosomal miR-193a regulates the cell-cycle cascade induced by epidermal growth factor receptor (EGFR) to dilute cell growth and proliferation. As an oncogenic factor, the methylation of the Wilms’ tumor 1 suppressor gene (WT1) promotor occurs in the early stage of BC. Overexpression of WT1 is positively correlated with the clinicopathological progress of BC. WT1 also takes part in EMT and MET equilibrium. Xie et al. have found that exosomal miR-193a blocks lesion formation and the metastatic tendency of BC cell lines by directly targeting WT1. In the meantime, they also found that ectopic expression of WT1 can partially rescue the metastasis-inhibitory effects exerted by upregulated miR-193a [97]. Therefore, exosomal miR-193a may exhibit biological activity through different proteins in different types of cells.

#### 3.2.7. miR-720

Exosomal miR-720 is attenuated in metastatic BC cell lines, partly demonstrating its tumor-inhibitory identity. By altering the ratio of epithelial markers (β-catenin and E-cadherin) and mesenchymal markers (vimentin, fibronectin, MMP-2, and N-cadherin), exosomal miR-720 impedes the EMT process to repress distant metastasis of BC cells [98]. Twist-related protein 1 (TWIST1), a fundamental molecule involved in various cellular bioactivities, is upregulated in several cancerous cells of the prostate, pancreas, and breast cells. The expression of TWIST1 augments the motility of epithelial cells. Activated by TWIST1, abnormally abundant HER2 appears in 20–30% case of BC, also responsible for more malignant traits and poorer survival rate [93,99]. By mediating the miR-720/TWIST1/HER2 axis, exosomal miR-720 represses the motion tendency of BC cells and metastasis cascade [98].

## 4. The Regulation of Exosomal microRNAs in the Stemness of BC Cells

CSCs sharing the same characteristics with normal stem cells, such as self-renewal and differentiation, are considered to be situated in the top level of tumor hierarchical organization. CSCs can give rise to phenotypically diverse tumor cell populations or remain undifferentiated with highly-differentiated potential, which imparts tumor heterogeneity and higher malignancy [100]. This subpopulation of tumor cells is the key to perpetuating the tumor. Different subsets of CSCs carrying various biomarkers according to their functions can promote metastasis and tumor recurrence [101].

Some cutting-edge researches have uncovered that exosomal miRNAs exert crucial roles in cancer stemness maintenance [102]. While some effects of exosomal miRNAs in breast cancer stem cells (BCSC) are unclear. Research on the underlying mechanism of exosomal miRNAs provides novel clues for more effective treatments. Next, we review the current understanding of the influence of the interaction between exosomal miRNAs and BC in metastasis and stemness.

Exosomal miRNAs have been demonstrated to involve in regulating cancer progressions, such as stemness and metastasis. By restraining the activity of phosphatase PTEN, exosomal miR-221/222 activates the Akt/NF-κB/COX-2 cascades and imparts stemness-like traits to BC cells [103]. While exosomal miR-143, miR-21, and miR-378e can strongly promote stemness [104]. EMT, a crucial process in tumor invasiveness and metastasis, is a developmental program that facilitates stemness acquisition and maintenance of non-transformed cells and CSCs [105]. Current studies reveal that exosomal miR-200 serves as a tumor suppressor, negatively regulating EMT and stemness by directly activating a series of inhibitory molecules, such as polycomb repressor complex protein B-lymphoma Mo-MLV insertion region 1 (Bmi1) and transcriptional repressor Zeb1/2. TET (Ten eleven translocation) family belongs to DNA demethylase, related to epigenetic modification and cancer cell differentiation. Exosomal miR-22 silences TET/miR-200 axis by inhibiting the TET family which in turn blocks the demethylation of the exosomal miR-200 promoter, contributing to increased EMT and stemness [106]. 

Exosomal miRNAs can also modify stemness by regulating the expression and function of BC stemness-related genes and the function of related elements. NOTCH1 is an important stemness-regulating gene for mammary stem cells and tumorigenesis. Exosomal miR-34a reduces NOTCH1 expression level to make asymmetric fate definition in BCs and weakens stemness in mammosphere cells [107]. Sox2 and sox9 are oncogenic transcription factors. Sox9 regulates stem cell properties and promotes the transformation from differentiated mammary epithelial cells to mammary stem cells, crucial in BC initiation and malignancy [108]. Sox2 recruits and cooperates with cofactors to constitute a complex binding to multiple genes’ promoters, which causes irregular self-renewal and increased formation of tumor subpopulation [39]. Exosomal miR-140 abundance is reduced in various cancers including BC. Exosomal miR-140 disturbs stem cell renewal and shrinks stem cell population by directly downregulating Sox2/Sox9 expression [39,109].

## 5. The Regulation of Exosomal microRNAs in the Angiogenesis in BC

Angiogenesis refers to form the neovessel from the preexisting vascular network, which is a highly-active and crucial process in oncogenesis [110]. It is well established that the more pathological angiogenesis occurs, the higher the probability of cancer cells migrating to the circulatory system and achieving distant metastasis. However, in recent years, numerous reviews have summarized and elucidated the mechanism of BC angiogenesis, but only a few have linked BC angiogenesis to exosomal miRNAs. Next, we will review the underlying mechanism of exosomal miRNA taking part in angiogenesis to impair or augment the metastasis of BC.

Exosomal miRNAs that increase the level of any pro-angiogenetic molecules can promote angiogenesis. By establishing an animal model of BC in mice, Kong et al. demonstrated that exosomal miR-155 regulates the von Hippel-Lindau (VHL)/hypoxia-inducible factor (HIF) axis and a cohort of downstream genes, such as interleukin 6 (IL6), vascular endothelial growth factor A (VEGFA), CD44, and pyruvate kinase isozyme type M2 (PKM2), to motivate angiogenetic cascades [111]. Elevated in BC patients, exosomal miR-132 increases the sensitivity of endothelial cells to VEGF by weakening the function of the RAS suppressor, reinforcing angiogenesis [112]. 

Anti-metastatic exosomal miRNAs regulate the network of angiogenetic-dependent factors to perturb angiogenesis. For example, exosomal miR-16 plays an anti-angiogenetic role by impeding the expression of VEGF [113]. Exosomal miR-503 modifies the tumor microenvironment and blocks the angiogenetic process by simultaneously perturbing the expression of two potent pro-angiogenetic molecules, fibroblast growth factor 2 (FGF2) and VEGFA [114]. After exposure to exosomal miR-100, the formation of human umbilical vein endothelial cells (HUVECs) networks is significantly reduced and the growth of cellular tubes is suppressed. Mechanically, exosomal miR-100 regulates the mTOR/ hypoxia-inducible factor-1 (HIF-1α)/VEGF axis to downregulate the activities of fundamental effectors involved in angiogenesis [115].

## 6. The Regulation of Exosomal microRNAs in Chemotherapy Resistance in BC

The recognized clinical treatment strategies for BC embrace specific chemotherapy, radiotherapy, and systemically surgical resection. Chemoresistance caused by repetitive and long-range usage of the same kind of drugs is the major obstacle for successful treatment, which accounts for almost 90% of the chemotherapy failures [116]. It is urgent to explore the underlying mechanism in drug resistance acquisition to reduce the recurrence rate. Numerous studies have uncovered the influence of exosomal miRNAs in resistance induction of BC. Here, we will review the signaling pathways targeted by exosomal miRNAs to regulate the drug sensitivity of BC cells. As shown in Table 1, we summarize the exosomal miRNAs and their targets in chemoresistance.

### 6.1. Topoisomerase Interactive Agents

#### 6.1.1. Doxorubicin (Adriamycin)

Doxorubicin is a commonly used chemotherapeutic drug that shows high activity against a variety of tumors. Early studies proved that doxorubicin can interfere with gene expression through intercalating into DNA double-helix and inhibiting enzymatic activities of topoisomerase II. It can enhance intercellular oxidative stress by promoting the conversion between oxygen and reactive oxygen species (ROS). The excessive stimulation of ROS will attack genomic and mitochondrial DNA, rendering them unstable. This is toxic and harmful for cell survival in doxorubicin-resistance groups [143]. Doxorubicin resistance has traditionally been a hot research topic in recent years. Studies have revealed that 66 miRNAs are increased and 309 miRNAs are decreased [144], which suggests that there is a high correlation between miRNA and doxorubicin resistance. Following, we focus on the concrete impacts of exosomal miRNA on doxorubicin resistance.

As an effective oncogene, Akt is homologous with the retroviral gene v-Akt. Akt and fundamental molecules are involved in its signal cascade to regulate DNA repair and cell proliferation. Highly activated in carcinogenesis, Akt suppresses apoptotic cell death program and improves cell survival by targeting a series of apoptotic effectors [120]. The Akt signaling network is significantly related to chemoresistance. Several studies have indicated that the variance of the Akt pathway by exosome microRNAs influences apoptosis induced by chemo-agents. Mechanically, exosomal miR-221-3p governs the activities of PI3K/Akt/phosphoinositide-3-kinase regulatory subunit 1 (PIK3R1) axis to facilitate the gain of doxorubicin resistance in cancerous cells [118]. Inversely, Exosomal miR-505 indirectly inhibits the Akt3 activities and induces apoptosis to restore doxorubicin sensitivity [120].

As a highly conserved process, autophagy refers to the capture of malfunctioning lipids, proteins, and organelles and carries them to the lysosome for degradation. The influence of autophagy is contentious. It supports cell survival in cytotoxic environments such as hypoxia and starvation. Furthermore, genotypic pressure caused by anti-tumor chemo-agents activates cytoprotective autophagy. However, lack of autophagy disturbs genetic stability and increases oxidative stress, which induces the initiation and progression of tumors [145]. Exosomal microRNAs are able to benefit from autophagy to modify cytotoxic effects induced by drugs and regulate cellular sensitization. Isoliquiritigenin (ISL), a powerful motivator of autophagy-lysosome cascades, is inhibited by exosomal miR-25 to imparts chemoresistance to Doxorubicin [119]. Inversely, by adjusting the interaction between downstream histone deacetylase 1 (HDAC1)/HDAC7/heat shock proteins 70 (Hsp70) K246, exosomal miR-34a inhibits autophagic cell death and weakens chemo-sensitizing potential of BC cells [121,122].

In fact, one exosomal microRNA may exert dual effects by targeting different effectors. The roles of exosomal miR-181a in doxorubicin resistance are dual by targeting different molecules. It increases the sensitivity to doxorubicin by modifying B-cell lymphoma 2 (Bcl-2) [123] and upregulates BCL2-Associated X (Bax) to weaken apoptosis under cytotoxic pressure from anti-cancer drugs [124].

Intriguingly, doxorubicin can suppress the expansion of the primary lesion, but it brings the risk of lung metastasis to some extent. The administration of doxorubicin induces a paracrine signal cascade which modifies the lung microenvironment to establish favorable conditions for future metastasis. Doxorubicin promotes cancerous cells to excrete IL-33 which subsequently induces the release of IL-13 from Th2 cells in the lung. Besides, doxorubicin accelerates the immunoinhibitory cells, myeloid-derived suppressor cells (MDSC), to coat with IL-13 receptors and synthesize exosomal miR-126a. After IL-13 derived from Th2 cells combined with its corresponding receptors on the surface of MDSCs, a vast array of exosomal miR-126a will be excreted and acts on Th2 cells, which, in turn, releases more IL-13 and induces more miR-126a. Exosomal miR-126a targets the S100A9 gene to facilitate the proliferation and survival of MDSC. This positive-feedback network exaggerates the influence of exosomal miR-126a and a cohort of inflammatory cells and molecules in angiogenesis, tumor growth, and metastasis in the lung, making adequate preparations for BC distant metastasis [125]. This finding lays a foundation for future studies to assess whether DOX-induced MDSC exosome miR-126a has an effect on BC cells and contributes to the development of chemoresistance in BC cells.

#### 6.1.2. Mitoxantrone

Mitoxantrone is a powerful topoisomeraseⅡ inhibitor, regarded as a cell-cycle nonspecific chemotherapeutic drug that suppresses the synthesis of genetic materials and blocks cell proliferation in every stage of the cell cycle [146]. Exosomal miR-328 was found to be associated with the cellular content of important breast cancer resistance protein (BCRP/ABCG2). Overexpressed exosomal miR-328 contributes to increased sensitization to Mitoxantrone of MCF-7/MX100 cells by downregulating BCRP/ABCG2 expression [126].

### 6.2. Platinum Analogs

#### Cisplatin

Cisplatin can directly bind to DNA, causing cross-linking, which generates double-strand breaks [147]. Numerous miRNAs are found to be dysregulated in these processes for the gain and loss of chemoresistance. Methyl-CpG binding protein 2 (MECP2), a methyl-binding protein involved in DNA transcription activities, is inhibited by exosomal miR-194 and exosomal miR-132 in cisplatin-resistant BC cell lines [127,128]. Besides, some selected candidate exosomal microRNAs can downregulate chemoresistance. Exosomal miR-24 promotes EMT and induces stemness by influencing FIH1 and BimL, enhancing cisplatin-resistance [121]. E2F1 governs G1/S transition and promotes the expression of downstream molecule ataxia-telangiectasia mutated (ATM). By directly perturbing E2F1 functions and indirectly repressing ATM, exosomal miR-302b causes irreversible cell-cycle arrest, deficiency in DNA repair, and promotes apoptosis after cisplatin administration [129].

### 6.3. Antimicrotubule Agents

#### Taxane

Taxane, which is a type of tricyclic compound, including paclitaxel (PTX) and docetaxel. The antitumor effects of taxane are generally attributed to inhibition of microtubule dynamics, leading to cell division defects [148]. 

Increasing studies demonstrate that the roles played by exosomal microRNAs are indispensable in the EMT which is crucial in the acquisition of multidrug resistance (MDR) phenotype [149]. By regulating EMT-associated molecules, such as Twinfilin 1 (TWF1) and Interleukin-11 (IL-11), exosomal miR-30c causes acquired PTX resistance [130]. In contrast, semaphorin 4C (Sema4C), a natural inducer of EMT, is inhibited by exosomal miR-125b, which reverse EMT phenotypes and augments sensitivity to PTX [132]. Re-expression of miR-200 family suppresses EMT by regulating ZEB1/ZEB2/E-cadherin, restoring sensitivity to microtubule-modulating drugs [131,133].

One of the hallmarks of cancer is the successful escape from programmed cell death [62]. Apoptosis-related molecules, such as BCL2 and BCL2 homologous antagonist/killer (BAK1) are the major effectors involved in this process, as well as take part in the exosomal microRNAs-mediated chemoresistance. By targeting BAK1, exosomal miR-125 interfering with and weakening PTX-caused apoptosis and cellular toxicity [131]. Inversely, the exosomal miR-16/BCL2 axis leads to an increased apoptotic program and imparts docetaxel sensitivity to cancerous cells [131].

### 6.4. Hormonal Agents

#### 6.4.1. Tamoxifen

Tamoxifen, an antagonist of the ER, suppresses estrogen-dependent growth [150]. As a kind of phosphatase, PTEN is a crucial inhibitor of PI3K, which precisely governs the PI3K/Akt/mTOR signal way. Loss or diminution of PTEN activities favors oncogenic progress and chemoresistance [151]. Exosomal miR-301 targets PTEN to cause the loss of response to tamoxifen therapy [134]. Exosomal miR-101 inhibits PTEN and activates Akt by targeting membrane-associated guanylate kinase (MAGI-2) to confer tamoxifen resistance to BC cells [135].

The balance between cell cycle arrest and cell death is usually inconstant in cancer treatment. Exosomal microRNAs can regulate the bioactivities of E2F, cyclins, and CDKs to coordinate cell cycle pace or make cell cycle chaotic to modify chemoresistance. MiR-320a modifies the cAMP-regulated phosphoprotein (ARPP-19)/estrogen-related receptor gamma (ERRγ) pathways to reduce the content of CCND1 and c-Myc, which re-sensitizes resistant cells to tamoxifen therapy [136].

Autophagy is an essential process to maintain homeostasis and is closely related to chemo-resistance. By directly inhibiting uncoupling protein 2 (UCP2)-mediated autophagy, exosomal miR-214 restores the response of cancerous cells to tamoxifen [137]. In addition, exosomal miR-415a weakens chemo-resistant potential to tamoxifen by adjusting the interaction of ERα and 14-3-3 protein zeta (14-3-3ζ), as well as inhibiting autophagy [138].

#### 6.4.2. Fulvestrant

Fulvestrant, by selectively reducing the estrogen receptor, and inhibiting aromatase, represses the synthesis of estrogen, sufficiently improving the survival rate of ER-positive BC patients [152]. Loss of sensitivity to tamoxifen happens in almost 50% of ER-positive BC patients [153]. Exosomal miR-221/222 is a pleiotropic and powerful oncogenic molecule, elevated after fulvestrant treatment. By dysfunction of transforming growth factor-beta (TGF-β) and β-Catenin and their downstream signaling network, exosomal miR-221/222 imparts fulvestrant resistance to BC cell lines [139]. Enhancer of zeste homolog 2 (EZH2), a tri-methylase for histone H3 lysine 27 to turn the chromosomes into a translation-inhibitory state, is bound by exosomal miR-101 to bring fulvestrant resistance to sensitive BC cells [131,135].

### 6.5. Monoclonal Antibodies

#### Trastuzumab

Trastuzumab suppresses irregular cell growth of BC by combining with HER2 whose gene sequence is amplified in 20% to 25% of BC patients [154].

The interaction between growth factors and their corresponding receptors is critical in metabolic activities, such as cell viability, inter- or intra-cellular communication, and response to therapy [155]. Transforming growth factor (TGF), EGFR and insulin-like growth factor 1 receptor (IGF1R) are the key molecules in these cellular processes. While exosomal miR-205-5p can sensitize BC cells to trastuzumab by targeting ERBB2 and regulating the P63/EGFR axis [140].

Besides, exosomal microRNAs can target some novel factors to influence chemoresistance. Exosomal miR-16 inhibits the formation of trastuzumab-resistant BC cell lines in vivo and vitro. Mechanically, miR-16 modifies chromatin assembly and regulates far upstream element-binding protein 1 (FUBP1) and cyclin J to enhance the cytotoxic effects of trastuzumab [141].

### 6.6. Others

#### Verapamil

Verapamil is a suppressor of the P-glycoprotein (P-gp), a transporter protein pumping anti-cancer chemo-agents out of cells [156]. Transfection of let-7 can decrease the sensitivity of verapamil. Exosomal let-7 can regulate the functions of RAS, estrogen receptor 1 (ESR1), caspase-3 (CASP3), and high mobility group AT-hook 2 (HMGA2), influencing cell-cycle, the transformation between EMT and MET, and hormone receptor expression [131].

In conclusion, the acquisition of drug chemoresistance by exosomal miRNAs can be generalized in the following ways. First, grab some critical signaling pathways to support cancer cellular independent growth. Second, promote EMT and stimulate the formation and expansion of the CSC population to dictate a more aggressive phenotype to cancer cells. Third, facilitate cell survival by the imbalance of autophagy/apoptosis and upregulation of anti-apoptotic molecules. Finally, yet importantly, dis-regulate drug transport to enhance the elimination of cytotoxic drugs from cells.

## 7. Exosomal microRNAs That Function as Prognostic Biomarkers for BC Patients

### 7.1. Exosomal microRNAs for Early-Stage Discovery of BC

Exosomal miRNAs work as effective instructors to tell us in which step is the oncogenic process going on and help doctors distinguish what kinds of BC are those states belonging to, which facilitates doctors to take further steps to copy with BC.

Exosomal miRNAs can predict the early occurrence of BC. Exosomal miR-221/222 almost penetrate every step of the oncogenesis of BC, which makes exosomal miR-221 an informative indicator for predictive BC origin, progress and treatment effects [157]. The increased concentration of miR-409-3p, miR-801, miR-127-3p, miR-652, and miR-148b in serum are found in stages I and II of BC, indicating their roles in detecting cancer early origin [158]. After amplification and quantitative detection of exosomal miR-21 by real-time PCR, the serum changes of exosomal miR-21 in 89 BC patients and 55 healthy controls were measured. The obvious increase of exosomal miR-21 in BC patients suggests its role in serving as biomarkers for early-phase BC, with 87.3% specificity and 87.6% sensitivity [159]. 

Besides, exosomal miRNAs are of great significance in predicting TNM staging, deterioration, and BC types with worse prognoses. The content of miR-197, miR-29b-2, miR-205, and miR-155 correspond to the lymph node involved in lesion metastasis (N3 versus N2) and tumor size (T3 versus T2). The occurrence of exosomal miR-205 and exosomal miR-155 suggests lesion metastases in the distance [160]. The elevation of exosomal miR-373 in TNBC is more significant than that in the luminal subtype whose presence demonstrates the hormone receptor-negative and triple-negative types of BC [46,161].

### 7.2. Exosomal microRNAs for Treatment Assessment of BC

Numerous studies demonstrate that the level changes of exosomal miRNAs before and after treatment can serve as the value index to reflect the therapy effect. Endothelial cells release exosomal miR-503 to regulate cell growth and migration of BC cell lines in vitro, reversing the aggressive phenotype of BC, which becomes elevated in patients after neoadjuvant treatment [162]. The anti-cancer drug can modify the quality and quantity of exosomal miRNAs, which informs us of the alteration in cancer status. Four overexpressed exosomal miRNAs, miR-376c, miR-27a, miR-155, and miR-376a, all turn back to normal concentration after neoadjuvant therapy, for further monitoring treatment progression [84]. After the administration of Trastuzumab, the concentration of exosomal miR-21 in HER2-positive patients is decreased, which suggests exosomal miR-21 can work as a Trastuzumab treatment assessment [163]. Elevated exosomal miR-454 level and reduced exosomal miR-374a/b indicate poor disease-free survival. Similarly, the increased content of some exosomal miRNAs (miR-210, miR-21, miR-454, miR-27a/b) and reduced expression of exosomal miR-155 are all typical markers for poor overall poor survival [164].

## 8. Exosomal microRNAs as Novel Targets for Targeted Therapy

**Figure 3:**
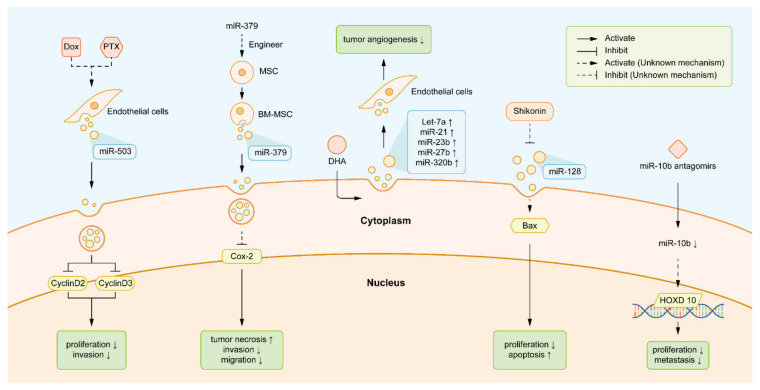
Exosomal miRNAs as novel targets for targeted therapy. The text legends from left to right are: (a) DOX and PTX induced endothelial cells to deliver more exosomal miR-503. The increased miR-503 interrupted the expression of CCND2 and CCND3, inhibiting BC cell proliferation and invasion consequently. (b) Engineering MSCs carried more exosomal miR-379 and released it into the tumor micro-environment by BM-MSCs, which interfered with the migration capacity of BC and induced the apparent tumor lesion necrosis. (c) DHA induced the elevated level of some exosomal miRNAs with negative effects on angiogenesis and tumorigenesis, such as let-7, miR-21, miR-23b, miR-27b, and miR-320b. (d) Shikonin inhibited the secretion of exosomal miR-128 in breast cancer cells, thus lifting its inhibition to the expression of the apoptotic gene Bax. Shikonin modified the interaction between exosomal miR-128 and Bax, subsequently promoting apoptosis. (e) MiR-10b antagomirs directly attenuated the bioactivities of exosomal miR-10b, which subsequently promoted the translation of HOXD10 and suppresses the migration of BC. Abbreviations: DOX: doxorubicin. PTX: paclitaxel. CCND2: cyclin D2. CCND3: cyclin D3. BC: breast cancer. MSCs: mesenchymal stem cells. BM-MSCs: bone marrow-derived mesenchymal stem cells. DHA: docosa-hexaenoic acid. HOXD10: Homeobox D10.

The roles of exosomal miRNAs in different stages of BC carcinogenesis have been reviewed in detail. We should combine the research value of exosomal miRNAs with their clinical value, making the research value better serve the clinical treatment. After clearly identifying the composition of miRNAs in different phases of BC, as shown in Figure 3, we can target specific miRNAs to treat various types of BC, timely change anti-cancer drugs to cope with chemoresistance, and monitor the real-time treatment response. 

Drugs that elevate the tumor-suppressive exosomal miRNAs can moderate the state of illness. Exosomal miR-503 is a powerful tumor- suppressor. After administration of neoadjuvant treatments, such as epirubicin and PTX, more exosomal miR-503 released from endothelial cells attenuates the aggressive traits of tumor [162]. Engineering mesenchymal stem cells (MSCs) carry more exosomal miR-379 and release the elevated level of miR-379, which contributes to the reduction in cell migration capacity and apparent BC tumor lesion necrosis [165]. Under the assistance of the Rab GTPase, Rab27A, docosahexaenoic acid (DHA) induces the elevated level of some exosomal miRNAs with anti-angiogenetic and tumor-inhibitory roles, such as let-7, miR-21, miR-23b, miR-27b, and miR-320b [166]. Sinomenine exerts its anti-tumor roles by regulating the miR-29/PDCD-4 axis, which subsequently suppresses cell viability capacity [167]. 

Simultaneously, the diminishment of tumor-promoting miRNAs is also the target task of anti-tumor medicine. Shikonin, an effective anti-tumor drug, inhibits the excretion of pro-tumorigenic exosomal miR-128 which facilitates the escape of BC cells from apoptosis [168]. Matrine decreases cell growth and enhances apoptosis by targeting the miR-21/PTEN/Akt pathway and downstream tumor-suppressive molecules [169]. MiR-10b antagomirs, an artificially-engineered anti-miRNA for systemic treatment, attenuates the activities of exosomal miR-10b and blocks the metastasis of BC cells to surround organs [56,59]. With superior efficacy in cancer treatment, doxycycline exerts powerful inhibitory effects on the PAR1/Akt/NF-κB/miR-17/E-cadherin pathway, which reverses aggressive phenotype and represses the motility of cancerous cells [170].

## 9. Discussion

Metastasis is the major cause of poor overall survival in BC patients. Distant metastatic lesions occur in almost half of BC patients administrated with hormone treatments and chemotherapy. Only 20% of the five-year survival rate happens in these patients [171]. The discovery and deep study of exosomal miRNA deepen an understanding of the potential mechanism of BC oncogenic progress. It has been indicated that exosomal miRNAs almost penetrate every step of various biological processes and takes indispensable roles [172]. Though the exploration of BC is quite advanced, only a small proportion of studies focus on the relationship between exosomal miRNA and BC metastasis in the last 5 years. In Table 2, we list the underlying mechanisms of different processes that exosomal miRNAs are involved in to contribute to metastasis and metastatic-related processes.

In this study, we have reviewed the latest findings of the roles played by exosomal miRNAs in BC metastasis and metastatic-related processes, including, migration, invasion, distal metastasis, stemness, and angiogenesis.

- MiRNAs involve controlling the aggressiveness of BC cells (such as miR-21, miR-10b, miR-1246, miR-373, and miR-17-5p).
- MiRNAs modify the stemness properties of BC cells (such as miR-22, miR-221/222, and miR-143).
- MiRNAs alter the angiogenetic process of BC (such as miR-155, miR-132, and miR-16).

In the next part, we review how exosomal miRNAs verify the sensitivity of BC cell lines to chemotherapy, and their instructive functions as biomarkers for diagnosis and prognosis (such as miR-221/222, miR-195, miR-1246, miR-484, and miR-182).

Demonstrated by different studies and conclusions, we tried to conclude some exosomal miRNAs carried dual effects on BC metastasis and metastasis-related processes. For example, different studies suggested miR-200 family (miR-200a, miR-200b, miR-200c, miR-429, miR-141) takes opposite roles in BC. The materials and methods employed in these experiments were different. Minh et al. transferred extracellular vesicles with miR-200 in different TNBC cell lines, 4TO7 and 4T1, observing the alteration of motion behavior of recipient cells [173]. Zhi-bin et al. used MDA-MB-231 cells for building a bone metastasis model, thereby uncovering the key modulator in the motion of BC cells to the bone micro-environment was miR-429/ZAB1/CRKL signaling network [86]. It can be deduced that the most likely reason for the discrepancy was the tumor microenvironment provided by different cell lines or inner interaction between miRNAs. More experiments that are comprehensive need to further investigate how the miR-200 family regulates the oncogenesis of BC.

At present, studies on whether and how exosomal miRNAs play a role in the treatment of BC are relatively abundant, and some of which, as we discussed earlier, give us a clearer understanding of the molecular mechanism of chemoresistance. In terms of radiotherapy, Tang et al. found that radiation-induced exosomal miR-208a enhanced the radioresistance of lung cancer cells, which first revealed the relationship between exosomal miRNAs and radiosensitivity [174]. Interestingly, recent experiments have confirmed that downregulation of miRNA-26b-5p enhances radiosensitization in lung adenocarcinoma cells [175]. Previous experimental results suggest that different exosomal miRNAs may exert opposite effects in different types of cancer cells. Unfortunately, as of this writing, no studies have directly elucidated the relationship between exosomal miRNAs and radiosensitivity of BC cells. We believe that this will be a promising area for future research.

As a highly conserved process, autophagy refers to assemble dysfunctional substances, such as damaged organelles and cytoplasmic proteins into bilayer autophagosomes. And after processing in lysosomes, components of autophagosomes are chosen to be degraded or recycled, which maintains cellular homeostasis [176]. Roles of autophagy in tumorigenesis are dual. At the beginning of metastasis, increased autophagy counteracts deleterious situations such as oxidative cytotoxicity and metabolic pressure, assisting the survival of cells and speeding up metastasis [177]. However, autophagy promotes inflammatory reactions and restrains the formation and expansion of primary lesions [178]. Some studies demonstrated the involvement of exosomal miRNAs in autophagy happening in BC. Biological activities of exosomal miR-92b induce autophagy in BC cells by downregulating the expression of EZH2 at mRNA level [179]. Instead, as a tumor-suppressive miRNA, exosomal miR-107 inhibits autophagy by mediating HMGB1 expression [180].

It was uncovered that autophagy can fuel tumor progress by weakening the negative influence of anoikis on cancerous cells [181]. Anoikis, as an indispensable tumor-inhibitory process, induces programmed cell death when sensing the detachment of cells from the native ECM, which is considered as an effective repressor in anchorage-free growth and abnormal detachment. Anoikis resistance refers to adherent-free growth and spontaneous EMT which are pivotal in facilitating distant metastasis and tumorigenesis. Elevated anoikis resistance imparts more aggressive traits to metastatic cells [182]. Erin N Howe et al. revealed that as a receptor tyrosine kinase situated in the cell surface, TrkB imparts increased anoikis resistance to BC cells after activation by its ligands NTF3. By directly targeting NTF3, exosomal miR-200 restores the anoikis sensitivity of cancerous cells of TNBCs [183]. However, at the time of writing, only a few studies have detailedly discussed the relationship of exosomal miRNA and anoikis in BC, which points out the direction for the next research.

BC cells are divided into five subtypes, HER2-overexpressing, basal-like, normal-like, luminal A, and luminal B. Due to their different clinicopathological features and prognostics, it is important in identifying candidate miRNAs to distinguish several subtypes for assessing the targeted clinical treatments. After analyzing clinical cases, exosomal miR-21 was found specifically elevated in several cancers, which effectively instructs TNBC [164]. Besides, it was discovered that exosomal miR-373, speeding up the tumorigenic processes, is significantly elevated in TNBC and ER/PR-negative patients compared with receptor-positive patients [184]. The content of exosomal let-7a, miR-1246, miR-10b, miR-21, and miR-122 all increase to varying degrees, which tells healthy patients apart from BC patients [161,185]. As consulting numerous literature, many studies have focused on how to distinguish healthy groups from BC patients, and tell TNBC apart from other types. Only a few studies clearly indicated the content difference between these five subtypes of BC and evidence to discriminate them under the assistance of exosomal miRNAs.

This comprehensive review provides a systematic overview to improve our understanding of the mechanisms behind the origin, maintenance, and progression of tumors. More importantly, new insights have helped to identify diagnostic, prognostic, and predictive markers at the molecular and cellular levels, paving the way for new therapeutic approaches.

## Figures and Tables

**Table 1:** Exosomal microRNAs take part in chemoresistance.

Exosomal microRNA	Drug	Signaling Axis and Pathway	Mechanism	Chemoresistance	References
miR-221-3p	Doxorubicin	miR-221-3p/PI3K/Akt/PIK3R1 pathway	Unknown	↑	[117,118]
miR-25	Doxorubicin	miR-25/ISL axis	Inhibit autophagy-lysosome cascades	↑	[119]
miR-505	Doxorubicin	miR-505/Akt3 pathway	Induce apoptosis under treatment	↓	[120]
miR-34a	Doxorubicin	miR-34a-HDAC1/HDAC7/HSP70 K246 axis	Inhibit proliferation and autophagic cell death	↑	[121,122]
miR-181a	Doxorubicin	miR-181a/Bax axis	Suppress apoptosis under cytotoxic pressure from drugs	↑	[123]
miR-181a	Doxorubicin	miR-181a/Bcl-2 axis	Induce programmed cell death	↓	[124]
miR-126a	Doxorubicin	The positive feedback in IL-33/IL13/miR-126a axis	Modify the tumor micro-environment for the distant metastasis	↑	[125]
miR-328	Mitoxantrone	miR-328/BCRP/ABCG2 pathway	Regulate drug disposition	↓	[126]
miR-194	Cisplatin	miR-194/MeCP2 axis	Influence epigenetic-related chemoresistance	↑	[127]
miR-132	Cisplatin	miR-132/MeCP2 axis	Influence epigenetic-related chemoresistance	↑	[127,128]
miR-24	Cisplatin	miR-24/FIH1/BimL pathway	Enhance EMT and stemness	↑	[121]
miR-302b	Cisplatin	miR-302b/E2F1/ATM pathway	Inhibit cell life cycle	↓	[129]
miR-30c	Taxane	miR-30c/TWF1/IL-11 pathway	Suppress EMT	↑	[130]
miR-125	Taxane	miR-125/BAK1 axis	Inhibit apoptosis induced by drugs	↑	[131]
miR-125b	Taxane	miR-125b/Sema4C axis	Reverse EMT phenotypes	↓	[132]
miR-200	Taxane	miR-200/ZEB1/ZEB2/E-cadherin pathway	Suppress EMT	↓	[131,133]
miR-16	Taxane	miR-16/BCL2 axis	Promote the apoptotic program	↓	[128,131,134]
miR-301	Tamoxifen	miR-301/PTEN axis	Unknown	↑	[134]
miR-101	Tamoxifen	miR-101/MAGI-2/PTEN/Akt cacscades	Unclear	↑	[135]
miR-320a	Tamoxifen	miR-320a/ARPP-19/ERRγ/ c-Myc/Cyclin D1 cascades	Make cancerous tissues re-sensitize to Tamoxifen	↓	[136]
miR-214	Tamoxifen	miR-214/UCP2 axis	Promote apoptosis	↓	[137]
miR-451a	Tamoxifen	miR-451a/Erα/14-3-3ζ axis	Block autophagy	↓	[138]
miR-221/222	Fulvestrant	miR-221,222/TGF-β/β-Catenin pathway	Support unlimited and anchorage-free growth	↓	[139]
miR-101	Fulvestrant	miR-101/EZH2 axis	Form a negative chromatin state	↓	[135]
miR-205-5p	Trastuzumab	miR-205-5p/ERBB2 axis	Unknown	↓	[140]
		MiR-205-5P/p63/EGFR pathway			
miR-16	Trastuzumab	miR-16/FUBP1/cyclin J axis	Enhance cytotoxic effects of drugs	↓	[141]
let-7	Verapamil	let-7/RAS/ESR1/CASP3/HMGA2 axis	Influence cellular response, regulate the expression of receptors and EMT progression	↑	[131,142]

**Table 2:** Different roles of exosomal microRNAs playing in BC progress.

Function		microRNA	Signaling Axis and Pathway	Mechanism	References
Promote	Invasion and migration of BC cells	miR-21	miR-21/PTEN/PI3K/Drg-1 axis	Contributes to the solid malignancy and hematological dissemination	[26,28,30]
			miR-21/maspin/PDCD4 axis	Facilitate the biological activities of tumor-suppressor genes	
		miR-10b	miR-10b/HODX10 axis	Block the formation of clones	[39,41,42]
			miR-10b/TBX5/PTEN, DYRK1A pathway	Unknown	
			miR-10b/syndecan-1 axis	Regulate cytoskeleton and E-cadherin expression	
		miR-1246	miR-1246/CCNG2 axis	Promote tumorigenesis	[43,45]
		miR-373	miR-373/CD44 axis	Facilitate the efficient migration and inhibit apoptosis	[46,47,48]
		miR-17-5p	miR-17-5p/HBP1/TCF/LEF/Wnt/β-catenin cascades	Unknown	[49]
		miR-96	miR-96/PTPN9/EGFR/STAT3/ErbB2 axis	Promotive the acquisition of increased motion ability	[51]
		miR-106b	miR-106b/FUT6 axis	Increase motion ability	[52]
	Distant metastasis of BC cells	miR-10b	Twist/miR-10b/HOXD10/RHoC axis	Inhibit the growth of the primary lesion and the formation of metastatic lesions	[57,58,59,60]
			miR-10b/E-cadherin axis	Unknown	
			miR-10b/NF1/HODX10/Rock/c-Jun axis	Influence cytoskeletal flexibility	
		miR-503	XIST/miR-503/STAT3/NF-κB/PD-L1 axis	Suppress local immunity	[61]
		miR-122	miR-122/PKM/GLUT1 axis	Provide the adequate nutrient availability	[63]
		miR-200	miR-200/Sec23a axis	Promote the formation of colonization	[64,65]
			miR-200/YAP1 axis		
		miR-105	miR-105/ZO-1 axis	Facilitate the dissemination and disaggregation	[24]
		miR-21	miR-21/LZTFL1/β-catenin/EMT axis	Enhance EMT, cell proliferation, and motility	[67]
	Stemness of BC cells	miR-22	miR-22/TET/miR-200 aixs	Promote clonal expansion	[106]
		miR-221/222	miR-221,222/PTEN/ Akt/NF-κB/COX-2 pathway	Unknown	[103]
		miR-143, miR-21, and miR-378e	miR-143,21,378e/Stemness markers (sox2, oct3/4, nanog)/EMT markers (zeb and snail)	Upregulate stemness biomarkers, promote anchorage-independent cell growth and EMT	[104]
	BC angiogenesis	miR-155	miR-155/VHL/HIF axis	Downregulate pro-angiogenetic substrates	[111]
		miR-132	miR-132/RAS/VEGF axis	Increases the sensitivity of endothelial cells to VEGF	[112]
Inhibit	Invasion and migration of BC cells	miR-564	miR-564/A cohort of genes (AKT2, GNA12, GYS1, and SRF)/PI3K/MAPK pathways	Arrest cell cycle progression	[69]
		miR-10a	miR-10a/PI3K/Akt/mTOR pathway	Disturb growth, motion and induce apoptosis	[70]
		miR-34c	miR-34c/GIT1 axis	Enhance intercellular adhesion	[71]
		miR-217	miR-217/KLF5 axis	Suppress cell survival and growth	[75]
		miR-100	miR-100/FZD8 axis	Upregulate tumor-inhibitory molecules and downregulate tumor-supportive molecules	[77]
			miR-100/Wnt/β-catenin signaling pathway		
		miR-1226-3p	miR-1226-3p/AQP5 axis	Modify cell interaction in various aspects	[78]
		miR-19a-3p	miR-19a-3p/FOSL1 axis		
		miR-19b	miR-19b/mucin 1 axis		
		miR-148b-3p	miR-148b-3p/TRIM 29 axis	Induce apoptosis and interrupt tumor progression	[81]
		miR-148a	unknown	unknown	[81]
		miR-503	miR-503/CCND2/CCND3 axis	Influence the aggressive capacity of cancerous cells	[84]
		miR-17/20	miR-17,20/E2F1/IL-8/CCND1 cascades	Block cell-cycle, inhibit the secretion of pro-metastatic substrates and disturb dissemination [86]	[85]
	Distant metastasis of BC cells	miR-429	miR-429/ZEB1/CRKL axis	Diminish bone metastasis	[86,87]
			miR-429/CrkL/MMP-9 axis	Protect the local bone from destruction and block distant metastasis	
		miR-124-3p	miR-124-3p/PDCD6 axis	Interfere with cell motility and viability	[88,89]
			miR-124-ep/E-cadherin/Vimentin/N-cadherin pathway	Block the EMT progression	
		miR-31	miR-31/a wide spectrum of genes (Fzd3, RhoA, ITGA5, and so on)	Coordinate the complicated invasion-metastasis cascades	[91]
		miR-124	miR-124/IL-11 axis	Modify the metastatic—microenvironment in bone	[92,93]
		miR-1	miR-1/Frizzled 7/TNKS2/Wnt/β-catenin signaling cascades	Impair metastasis and subsequent tumorigenesis	[94,95]
			miR-1/BCL2 axis	Induce apoptosis	
		miR-193a	miR-193a/EGFR	Inhibit cell motility and metastatic clonization	[97]
			miR-193a/WT1 axis		
		miR-720	miR-720/epithelial markers (β-catenin and E-cadherin)/mesenchymal markers axis	Impede EMT	[98]
			miR-720/TWIST1/HER2 axis	Prevent the distant seeding and disaggregation	
	Stemness of BC cells	miR-34a	miR-34a/NOTCH1 axis	Inhibit stem cell compartment	[107]
		miR-140	miR-140/Sox2/Sox9 axis	Disturb stem cell renewal and shrink stem cell population	[39,109]
	BC angiogenesis	miR-16	miR-16/VEGF axis	Diminish pro-angiogenetic molecules	[113]
		miR-503	miR-503/FGF2/VEGFA axis	Perturbing the expression of potent pro-angiogenetic molecules	[114]
		miR-100	miR-100/mTOR/HIF-1alpha/VEGF axis	Reduce the fundamental effectors in angiogenesis	[115]

## Data Availability

The data presented in this study are available on request from the corresponding author.
